# Effective *in vivo* treatment of acute lung injury with helical, amphipathic peptoid mimics of pulmonary surfactant proteins

**DOI:** 10.1038/s41598-018-25009-3

**Published:** 2018-05-01

**Authors:** Ann M. Czyzewski, Lynda M. McCaig, Michelle T. Dohm, Lauren A. Broering, Li-Juan Yao, Nathan J. Brown, Maruti K. Didwania, Jennifer S. Lin, Jim F. Lewis, Ruud Veldhuizen, Annelise E. Barron

**Affiliations:** 1 Department of Chemical and Biological Engineering, Northwestern University, Evanston, Illinois USA; 2 Lawson Health Research Institute, Department of Physiology and Pharmacology, The University of Western Ontario, London, Ontario, Canada; 3 Department of Chemistry, Northwestern University, Evanston, Illinois USA; 4 Department of Bioengineering, Stanford University, School of Medicine, Stanford, California, USA

## Abstract

Acute lung injury (ALI) leads to progressive loss of breathing capacity and hypoxemia, as well as pulmonary surfactant dysfunction. ALI’s pathogenesis and management are complex, and it is a significant cause of morbidity and mortality worldwide. Exogenous surfactant therapy, even for research purposes, is impractical for adults because of the high cost of current surfactant preparations. Prior *in vitro* work has shown that poly-*N*-substituted glycines (peptoids), in a biomimetic lipid mixture, emulate key biophysical activities of lung surfactant proteins B and C at the air-water interface. Here we report good *in vivo* efficacy of a peptoid-based surfactant, compared with extracted animal surfactant and a synthetic lipid formulation, in a rat model of lavage-induced ALI. Adult rats were subjected to whole-lung lavage followed by administration of surfactant formulations and monitoring of outcomes. Treatment with a surfactant protein C mimic formulation improved blood oxygenation, blood pH, shunt fraction, and peak inspiratory pressure to a greater degree than surfactant protein B mimic or combined formulations. All peptoid-enhanced treatment groups showed improved outcomes compared to synthetic lipids alone, and some formulations improved outcomes to a similar extent as animal-derived surfactant. Robust biophysical mimics of natural surfactant proteins may enable new medical research in ALI treatment.

## Introduction

Lung surfactant is a complex lipid-protein mixture that coats alveolar surfaces and reduces surface tension at the air/liquid interface, enabling normal respiratory function^1^. The absence or inactivation of lung surfactant results in lung collapse and respiratory distress syndrome (RDS)^2,3^. The lack of surfactant related to infant RDS (IRDS)^4^ is routinely treated with animal-derived exogenous surfactant, which improves outcomes^1,4,5^. Acute lung injury (ALI) is a more complex disease resulting from a diverse set of etiologies^2,6,7^. Lung inflammation and alterations to endogenous surfactant result in hypoxemia and decreasing pulmonary function^2,8^.

Progressive lung dysfunction results from alterations in the lipid profile and surfactant-associated protein amounts, or changes in the relative amounts of surfactant aggregate forms^9,10^. Animal and clinical pilot studies suggest a sound rationale for using surfactant replacement therapy to mitigate some of these changes in a subset of ALI patients^2,7^ but unfortunately, larger Phase III clinical trials have had variable outcomes^11–13^. Factors contributing to inconsistent clinical results include: 1) mode, dose, and timing of surfactant delivery, 2) post-administration ventilation strategy, and 3) surfactant characteristics^2^. Reduced alveolar delivery of the surfactant has also been suggested as a contributing factor based on recent computer modeling^14^.

The biophysical activity, biostability/bioavailability, metabolic fate, and availability and cost of exogenous surfactant preparations impact their suitability for treatment of ALI patients. “Biomimetic” synthetic surfactants, designed to emulate features of animal-derived surfactant, are an emerging class of treatments that could provide benefits for ALI patients, as well as address the resource limitations inherent in animal-derived medications. Lipid and protein components are essential to animal-derived surfactant function. Lung surfactant proteins B and C (SP-B and SP-C) are two hydrophobic proteins essential for the adsorption and spreading of the surfactant film at the air-liquid interface^15^. These proteins have proven challenging to synthesize in active form (either chemically or recombinantly), thus most research has focused on development of simpler analogs of these proteins that replicate key features for bioactivity^16–18^.

Biomimetic surfactants comprising lipids and mimics of SP-B or SP-C made with helical, poly-*N*-substituted glycines (peptoids) exhibit promising *in vitro* biophysical activity^19–23^. Peptoids are non-natural compounds with a polypeptide backbone, but with side chains appended to the backbone nitrogens rather than the α-carbons^24^. Peptoid structure is advantageous for biomedical applications; their highly stable structure is protease-resistant, thus improving biostability and bioavailability while reducing immunogenic response; and they can be designed to form stable amphipathic helices^25^.

**Figure 1:**
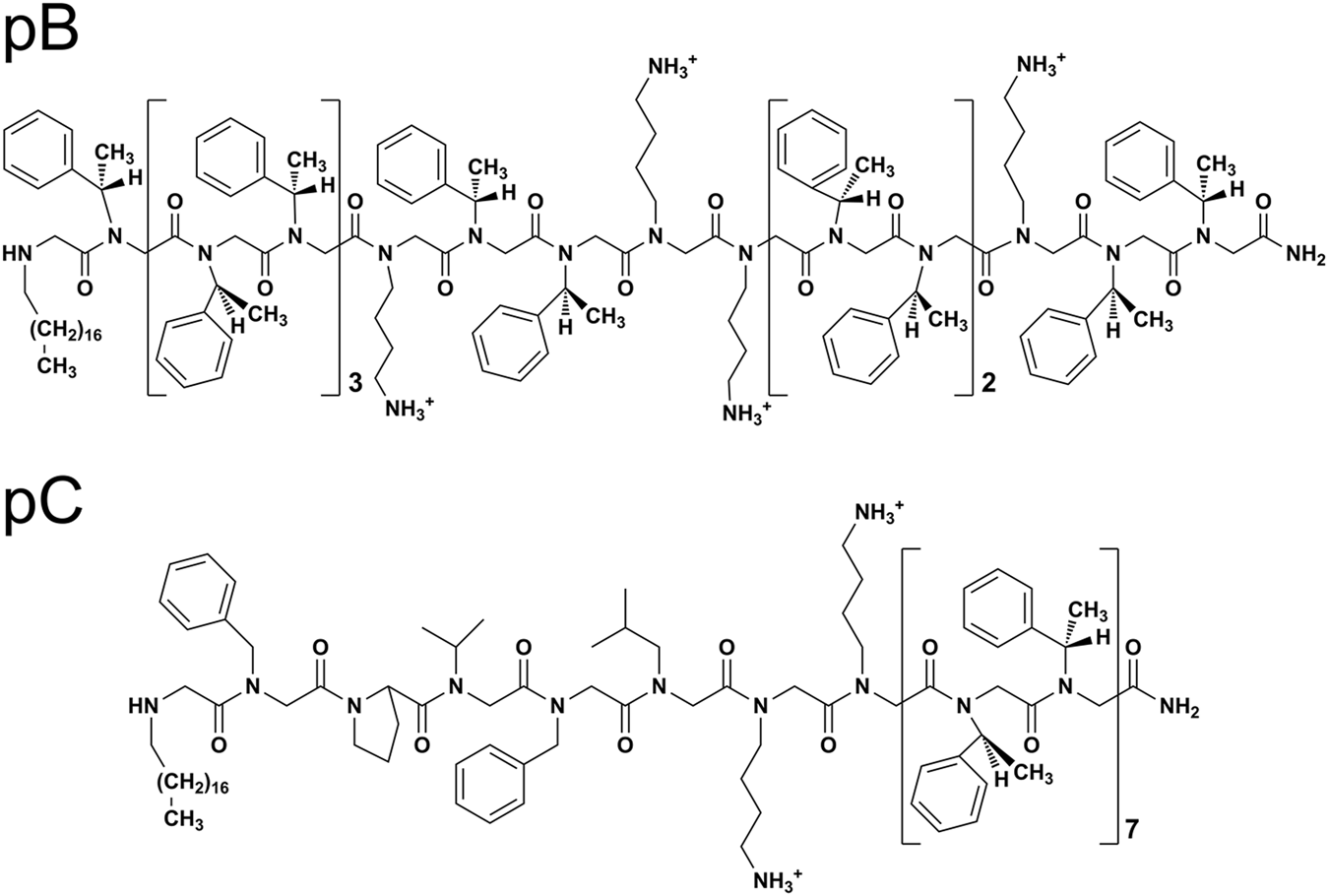
Chemical structures of peptoid-based mimics of SP-B (**pB**)^26^ and SP-C (**pC**)^27^. The eight *N*-terminal residues of **pC** contain side chains that are analogous to SP-C_5–12_, and the remaining 14 aromatic hydrophobic residues form a helix that mimics the membrane spanning, hydrophobic helix of native SP-C. The *N*-terminal octadecyl amine of **pC** is a motif intended to mimic the post-translational modification of palmitoylated residues 5 and 6 in human SP-C. The mimic **pB** was designed to emulate the insertion region and helical amphipathic patterning of SP-B_1-25_, with the added feature of an *N*-terminal octadecylamine.

We examined the ability of peptoid-based biophysical mimics of SP-B and/or SP-C, termed **pB** and **pC**, to mitigate deleterious physiological and biochemical responses associated with ALI. **pB** and **pC** (Fig. 1) were designed with sequence attributes that mimic the overall hydrophobicity, amphipathicity, and helical structures of SP-B and SP-C, respectively^26,27^. Both molecules are *N*-terminally C18-alkylated to mimic the palmitoyl moieties of natural SP-C, a feature known to improve *in vitro* surface activity^26,27^. The design of the SP-B mimic is more loosely biomimetic of the natural protein, while the SP-C mimic is more similar to the natural protein. Our hypothesis was that peptoid-enhanced surfactants could demonstrate *in vivo* efficacy in an animal model of ALI that matches or exceeds that of animal-derived surfactant.

## Methods

### Peptoid Synthesis and Surfactant Preparation

Peptoids were synthesized according to previously published protocols^19,26,28^. Tanaka lipids (DPPC:POPG:PA 68:22:9 [wt]) were prepared and dried^26,29^, with 2 mol% peptoid (1 mol% each for **pB/pC**). On the day of the experiment, sterile saline was added to the dehydrated surfactant mixture to make a homogeneous, fluid lipid-peptoid surfactant suspension at a concentration of 25 mg/mL. Additional details can be found in the Supplementary Information.

#### Animal Experiments

Procedures were approved by the Animal Use Subcommittee (AUS) at University of Western Ontario, London, Ontario, Canada, according to the Canadian Council on Animal Care (CCAC). All experiments were performed in accordance with relevant guidelines and regulations. Sprague-Dawley rats (360–410 g) (Charles River, St. Constant, PQ, Canada) were weighed, anesthetized by intraperitoneal (i.p.) injection (75 mg/kg ketamine hydrochloride, 5 mg/kg xylazine, sterile 0.15 M saline), and given 0.05–0.1 mg/kg of buprenorphine subcutaneously. Right carotid artery access *via* isolation and cannulation permitted blood gas sampling, measuring vital signs, and instilling fluids (0.15 M saline, 0.5 mL/kg/hour), while similarly obtained jugular venous line access allowed administering of ~1.0 mg/kg/min propofol and fluids. Following tracheostomy and endotracheal tube placement, pancuronium bromide (1 mg/kg i.v.) was administered to inhibit spontaneous movement. Ventilator settings were: tidal volume, 8 mL/kg; positive end expiratory pressure (PEEP), 5 cm H_2_O; respiratory rate, 55–60 breaths/minute; and fraction of inspired oxygen (FiO_2_), 1.0 (volume-cycle mechanical rodent ventilator, Harvard Instruments, St. Laurent, PQ, Canada; airway pressure monitor, Caradyne Ltd, Indianapolis, IN). Initial inclusion criterion was baseline average blood oxygen level (PaO_2_) >400 mmHg.

Whole lung lavage was performed^30–33^. After ventilator removal, 0.15 M NaCl (10 mL, 37 °C) was instilled/withdrawn from the lungs, followed by mechanical ventilation. Lungs were lavaged four times, 5 minutes apart preceding a blood gas measurement. Study inclusion required PaO_2_ <120 mmHg. Non-inclusive animals were re-lavaged until the inclusion criterion was satisfied^30^.

Animals were randomized into five treatment groups: 1) bovine lipid extract surfactant (BLES, BLES Biochemicals, London, Ontario, CA, the more widely utilized of the two bovine extracted surfactants available in Canada^34,35^ and the market leader in India and several countries outside of the USA and Europe) (positive control, n = 7), 2) **pB** (n = 6), 3) **pC** (n = 7), 4) **pB**/**pC** (n = 7), 5) Tanaka lipids (negative control, n = 5). Note that as a commercially available surfactant BLES was used as is while additional preparative work, described above, was needed to make the other surfactant treatments prior to dosing. After ventilator removal, upright animals were instilled with a 50 mg/kg surfactant bolus endotracheally *via* syringe, and then a 3-mL air bolus that ensured distribution to distal regions. This surfactant dosage was the same for all treatment groups. Ventilation and monitoring occurred for 2 hours, with blood gas sampling at semi-regular timepoints. Recovery vital signs were monitored for adequate perfusion, as was anesthetic state. Measured physiological responses included PaO_2_, blood pH, shunt fraction, Alveolar-arterial (A-a) gradient, and peak inspiratory pressure (PIP). Post-experiment, animals were euthanized *via* sodium pentobarbital, exsanguinated, the chest wall opened, lung-lavaged five times, and total lavage volume was recorded^36^.

### Surfactant Analysis

After broncheoalveolar lavage fluid (BAL) centrifugation (150 *g*, 10 minutes), 5 mL of supernatant “Total Surfactant (TS)” was aliquoted for further analysis, and the remainder centrifuged (40,000 *g*, 15 minutes) to separate supernatant Small Aggregates (SA) from pellet. Resuspended pellets (in 2 mL saline) produced Large Aggregates (LA). Aliquots were extracted (Bligh/Dyer method^37^), phospholipids quantified (Duck-Chong phosphorous assay^38^), and BAL total protein content determined (micro-BCA protein assay, Pierce Biotechnology, Rockford, IL).

### Statistical Analysis

Presented data are means ± SEM and were analyzed *via* one-way ANOVA using the Tukey-Kramer method (p < 0.05).

### Data Availability

The datasets generated during and/or analyzed during the current study are available from the corresponding author on reasonable request.

## Results

### Physiological Responses

**Figure 2:**
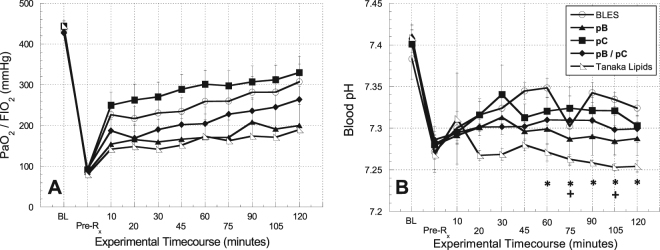
Physiological indicators of pulmonary gas exchange function over time. (**A**) PaO_2_/FIO_2_ and (**B**) Blood pH over the time course of the experiment. Error bars indicate the standard error of the mean (SEM). Statistical significance indicators: * indicates p < 0.05 between BLES treatment group and Tanaka Lipids; + indicates p < 0.05 between pC treatment group and Tanaka Lipids.

In general, the surgical procedure was well-tolerated by the animals. Three animals died during the procedure, and 13 animals did not meet the study inclusion criteria. For the 32 animals included in the study, the average baseline blood oxygen level (PaO_2_), normalized to the fraction of inspired oxygen (FiO_2_) (1.0 throughout all experiments), of 435.7 ± 4.9 mm Hg was reduced to 88.3 ± 2.5 mm Hg post-lavage, reflective of surfactant deficiency (Fig. 2A). Similarly, the average baseline peak inspiratory pressure (PIP) of 12.0 ± 0.3 cm H_2_O was increased to 20.8 ± 0.4 cm H_2_O post-lavage. The average blood pressure and heart rate for each treatment group over the time course of the experiments are shown in Supp. Figure S1.

The average PaO_2_/FIO_2_ as a function of time is shown for each treatment group in Fig. 2A. The immediate response to surfactant treatment is reflected in changes from the pre-treatment (Pre-Rx) condition to the 10-minute data time point. The ability of surfactant treatments to sustain a response was gleaned by comparing the 10-minute time point data to conditions at the end of the two-hour observation period. Animals treated with the positive control BLES (p < 0.001), **pC** (p < 0.0005), and **pB/pC** (p < 0.007) showed a statistically significant, immediate improvement in oxygenation upon treatment. The immediate improvements demonstrated by the negative control Tanaka lipids group (p < 0.23) and **pB** group (p < 0.07) were not significant. Treatment with BLES (p < 0.16), **pC** (p < 0.15), and **pB/pC** (p < 0.11) also demonstrated better sustained oxygenation throughout the two-hour observation period compared with the **pB** (p < 0.47) and Tanaka lipid (p < 0.60) treatment groups. Animals in the **pC** treatment group were correlated with the highest arterial blood oxygenation levels throughout the study.

The blood pH as a function of time is shown for each treatment group in Fig. 2B. Comparing baseline to pre-treatment conditions, pulmonary lavage caused a significant (p < 0.005) and uniform lowering of the blood pH in all treatment groups. On average, the highest blood pH outcome was achieved by the BLES treatment group, which was found to be statistically different (p < 0.05) from the Tanaka lipids treatment group at t > 45 minutes. Among peptoid-enhanced surfactants, treatment with **pC** exhibited the most complete return to baseline conditions; the blood pH of this treatment group was statistically different (p < 0.05) from the Tanaka lipids treatment group at 75 and 105 minutes.

**Figure 3:**
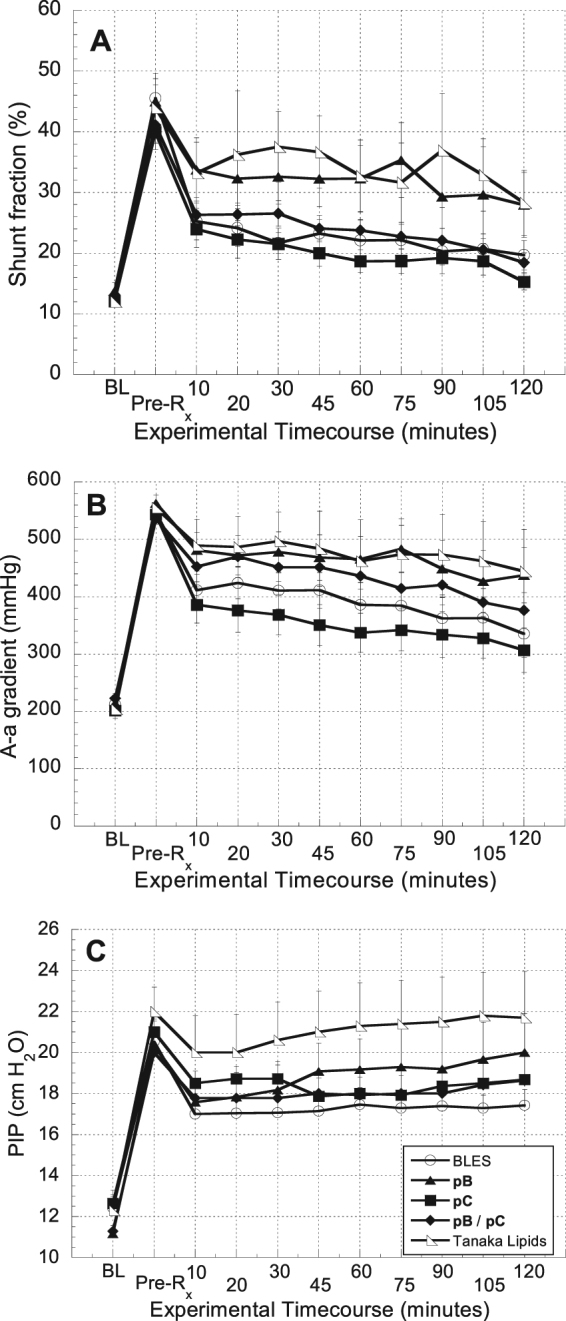
Physiological indicators of pulmonary function. (**A**) Shunt fraction (**B**) A-a gradient, and (**C**) Peak inspiratory pressure (PIP) over the time course of the experiments. Error bars indicate the standard error of the mean (SEM).

Figure 3 displays three additional indicators of pulmonary function, including shunt fraction, A-a gradient, and PIP. Ten minutes after treatment, the shunt fraction decreased significantly for the BLES (p < 0.0005), **pC** (0.001), and **pB/pC** (p < 0.01). The further decrease in shunt fraction observed from the 10-minute time point until the end of the two-hour observation period was statistically significant for the **pC** (p < 0.05) and **pB/pC** (p < 0.05) treatment groups. As shown in Fig. 3A, the **pC** treatment group was shown to be statistically different (p < 0.05) from the **pB** and Tanaka lipids treatment groups at selected timepoints. The A-a gradient data shown in Fig. 3B exhibits a statistically significant, immediate response for all treatment groups (p < 0.05) except Tanaka lipids (p < 0.20). The **pC** treatment group resulted in the most significant immediate decrease (p < 0.0003), but the **pB**/**pC** treatment group resulted in the best sustained response from the 10-minute time point throughout the observation period (p < 0.1). The PIP data shown in Fig. 3C demonstrated statistically significant immediate improvement at 10 minutes for BLES (p < 0.05) and **pC** (p < 0.01) (**pB** and **pB**/**pC** exhibited p < 0.1). The Tanaka lipid treatment group immediate improvement was not significant (p < 0.2). No treatment groups demonstrated sustained improvement of PIP throughout the 2-hour observation period.

### Surfactant Pool Evaluation

**Figure 4:**
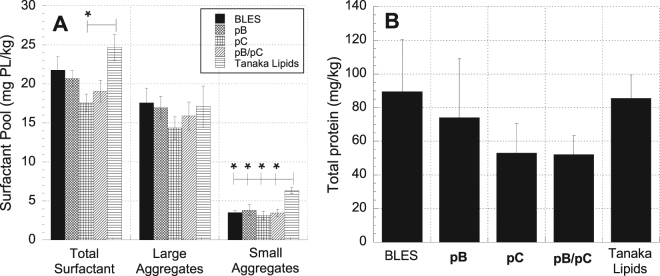
Surfactant pool characterization in broncheoalveolar lavage (BAL). (**A**) Average amounts of total surfactant, large aggregates, and small aggregates in BAL. (**B**) Average total protein content in the BAL of each treatment group. Error bars indicate the standard error of the mean. Statistical significance indicators: * indicates p < 0.05 for the difference between the designated group and TL alone group.

Since the efficacy of surfactant may be influenced by its metabolism within the airspace, phospholipid pools and total protein content in the broncheoalveolar lavage (BAL) fluid from each animal were evaluated at the end of the ventilation period. The average amount of total surfactant (TS), large aggregates (LA), and small aggregates (SA) obtained from the BAL of each treatment group is shown in Fig. 4A. While there was no statistically significant difference between the large aggregate contents of the treatment groups, the amount of small aggregates was higher in the Tanaka lipid treatment group than in any other (p < 0.05). The average amount of total surfactant was highest for the Tanaka lipids treatment group, and statistically higher (p < 0.05) than the **pC** treatment group. The average total protein content of the BAL for each treatment group is shown in Fig. 4B. The data show that there was no statistically significant difference in the total protein content among the various treatment groups.

## Discussion

While the extensive alveolar network and capillary vasculature of the pulmonary parenchymal tissue are critical to achieving efficient gas exchange, these delicate structures are highly susceptible to systemic pathogens and environmental toxins^7^. A broad spectrum of direct pulmonary insults and indirect systemic maladies results in lung surfactant deficiency and dysfunction, which leads to ALI^2,7,39^. There is currently no cure for ALI, and while exogenous surfactant treatment as part of a multimodal therapy has been shown to mitigate symptoms of the disease for a subset of patients^2,7^, outcomes of clinical trials have been varied^2,7,13^. We propose that peptoid-based, biomimetic surfactant replacements provide a novel technology platform with characteristics amenable to the treatment of ALI. In this inaugural study designed to investigate the *in vivo* efficacy of peptoid-based surfactants, we demonstrate, using the lung lavage model of ALI, that these lung surfactant replacements can improve physiological and biochemical outcomes to an equivalent extent as treatment with animal-derived surfactant. Peptoid-enhanced surfactant preparations demonstrated statistically significant immediate (within 10 minutes of treatment) and/or sustained (10 minutes–2 hours) improvements in PaO_2_/FIO_2_, shunt fraction, A-a gradient, and PIP. This is an encouraging result for biomimetic surfactants as it marks the first reporting of peptoid-enhanced surfactants demonstrating *in vivo* efficacy. In addition, for two measurements, PaO_2_ and A-a gradient (Figs 2A and 3B, respectively), the pC preparation appeared to perform slightly better than BLES based on the observed values, highlighting the potential of peptoid-based surfactants.

While peptoids have been shown previously to mimic the *in vitro* surface activity of SP-B^20,22,23,26^ and SP-C^19,21,27^, this study evaluated the *in vivo* efficacy of peptoid-based SP-B and SP-C mimics formulated separately and in combination. Tanaka lipids^29^ were selected as the lipid carrier for these synthetic formulations based on its similarity to the lipid/fatty acid component of natural surfactant^29^ and superior *in vitro* surface activity compared to other lipid formulations^23^. Though peptoid-enhanced surfactants contain a higher amount of protein mimic (~10 wt%) relative to the quantities of SP-B and SP-C found in extracted surfactant (~0.5–3 wt% each), this is reasonable because peptoids **pB** and **pC** (20 and 22 residues, respectively) represent only a portion of the natural proteins’ structures (79 and 35 residues for SP-B and SP-C, respectively). Moreover, the therapeutic dose of peptoid-enhanced surfactant has not been optimized in this study. The 50 mg/kg dose was chosen for this study based on previous experimental work in this animal model with BLES^32^. Additionally, the intent of this study is to test activity of the surfactants *in vivo* rather than clinical efficacy in an animal model. A reduced BLES dosage may highlight differences among the preparations, allowing the tested surfactants an opportunity to demonstrate superiority, inferiority, or equivalency whereas a higher BLES dose would only be a test for inferiority or equivalency. It is also notable that synthetic surfactants containing functional protein (peptide-based) mimics are not unprecedented. Lucinactant® (Surfaxin)^40^ from Discovery Labs contains KL4 (sinapultide), a 21mer peptide comprised of lysine and leucine, that was approved for use in the United States by the Food and Drug Administration in 2012 as a treatment for RDS in premature infants. Previous work has shown that **pB** and **pC** - formulated separately - showed improved *in vitro* surface activity compared to KL4 in a Tanaka lipid formulation^26,27^. Another synthetic surfactant formulation consisting of phosphatidylcholine, phosphatidylglycerol, and peptide analogs of SP-B and SP-C, designated CHF5633, is being developed by Chiesi Farmaceutici^17^ and compares favorably to animal-derived surfactant^41,42^. Most recently, this synthetic surfactant has been tested successfully in a Phase I clinical trial of preterm infants with RDS^43^. The success of Lucinactant® and the ongoing clinical development of CHF5633 is encouraging for the therapeutic potential of peptoid-enhanced surfactants.

We utilized a lung lavage model of surfactant deficiency in adult rats, a model that is well-characterized and has previously been shown to respond to animal-derived surfactant preparations^36,44^. The average heart rate and blood pressure of the various treatment groups showed no notable difference among any of the groups (Figure S1). The average post-lavage decrease in the PaO_2_ and increase in PIP showed that pulmonary gas exchange and lung compliance were significantly and uniformly damaged, a condition associated with ALI. While this animal model of surfactant deficiency does not capture all aspects of the pathophysiology associated with ALI (*i.e*. surfactant alterations) and is not intended to model IRDS, this simple, established protocol was deemed well-suited for direct comparison of surfactant preparations.

Previous work with this model has shown that animals which receive no treatment (air bolus only) have low PaO_2_/FIO_2_ values throughout the experiment (~100 mmHg)^30^, indicative of surfactant deficiency. Similar low values were observed in the animals in this study after lavage and prior to surfactant treatment as would be expected. PIP values for an air bolus only control group were consistently greater than 19 cmH_2_O^30^. Again, we see comparable values in our studies after lavage but prior to treatment where the PIP values were ~20 cmH_2_O. The five physiological responses measured in this study consistently showed that the negative control treatment group (Tanaka lipids) resulted in the least improvement in pulmonary function. Figures 2 and 3 show that the ‘Tanaka lipids alone’ formulation neither achieved the same initial degree of recovery, nor effectively maintained activity throughout the observation period. The notable and consistent improvement in physiological response to peptoid-enhanced Tanaka lipid formulations compared to the lipid carrier alone provide evidence for the bioactivity of peptoids to effect improved outcomes using a lung lavage model of ALI.

We have demonstrated the *in vivo* efficacy of peptoid-enhanced surfactant to mitigate the conditions associated with ALI, and this is a significant result because biomimetic exogenous lung surfactants afford several advantages over animal-derived surfactant replacements. The high cost of natural surfactant coupled with the large quantities required to treat adults for ALI can make treatment prohibitively expensive. Moreover, the use of a biomimetic surfactant avoids the risk of immune response that is inherent with animal-derived products. Biomimetic surfactants also offer the possibility of a “designer” treatment that could be customized to mitigate specific types of surfactant dysfunction or deactivation induced by the myriad of clinical maladies that result in ALI^45^. It is conceivable, for example, that additives could be included in a synthetic formulation, not only to improve surface activity, but also to prevent surfactant inhibition, regulate surfactant homeostasis, and control inflammatory response^7,18,45^. Finally, peptoids designed as biomimetics specifically exhibit secondary structure that makes them less prone to aggregation, which can result in enhanced shelf-life and facilitates synthesis and purification^46–48^.

Whereas all peptoid-containing surfactants improved physiological lung function, there were differences in the responses to the individual preparations. The formulation **pC** demonstrated a more significant initial improvement in physiological responses and exhibited sustained benefit throughout the recovery period, compared to the other preparations. The **pB** treatment group, however, consistently had a less significant impact on measured outcomes, and on average appeared only marginally better than Tanaka lipids.

A second observation regarding the responses of the individual preparations lies in comparing the performance of **pB** and **pC** formulated separately to that of the **pB**/**pC** combination formulation. By all physiological measures, the **pC** treatment group achieved a more favorable outcome than did the **pB** treatment group. Interestingly, however, the **pB**/**pC** group achieved the best sustained response in PaO_2_/FIO_2_, shunt fraction, and A-a gradient. The literature provides mixed evidence as to whether natural SP-B and SP-C or other SP-B/SP-C mimics interact synergistically, although most of the research suggests the presence of both SP-B and SP-C (or their mimics) is beneficial^49–54^. The degree of synergistic or additive surface-active behaviors of these mimics is ostensibly related to their underlying mechanisms of action. Moreover, it is likely that variability in the dynamic *in vivo* environment and lipid composition can influence the extent to which proteins and protein mimics interact. Because the synergistic interaction of protein mimics is dependent on both their chemical structures and the conditions *in vivo*, it is difficult to generalize observations relevant to a particular system. In this study, however, co-dosing **pB** and **pC** in the **pB**/**pC** formulation enabled a better sustained response in some physiological outcomes over the two-hour recovery period.

The way in which exogenously administered surfactant is metabolized is another factor that can influence its efficacy. Surfactant delivered to the airspace can subsequently be taken up by alveolar type II cells for recycling or by alveolar macrophages for degradation. In addition, within the airspace, exogenous surfactant can be converted from the active large aggregates to inactive small aggregates. These processes would all impact the efficacy of the exogenous material, and the surfactant pool characterization at the end of the ventilation period provided some insight into these effects. The data showed that the Tanaka lipids treatment group had a larger total surfactant pool than any other group and was statistically different from that of the **pC** treatment group (Fig. 4A). This difference could be due to disparate surfactant uptake rates for the various surfactant preparations. Because the Tanaka lipid formulation contains no proteins or protein mimics, it is possible that it may not be as readily taken up by type II cells. The rate of conversion from large to small aggregates within the surfactant pool has also been shown to increase under conditions pervasive in an injured lung: 1) increased protease activity^55,56^, 2) altered surfactant composition^57,58^, and 3) dynamic changes in surface area due to mechanical ventilation^59,60^. Injured lungs, therefore, often exhibit an increased amount of total surfactant and a concomitant increase in the less surface-active small aggregates^2,9,10,61^. Figure 4A shows that indeed the small aggregate component of the BAL from the Tanaka lipid treatment group was statistically greater than that of any other group. The increase in total surfactant of this group appears to be due to primarily an increase in the less active small aggregates.

The results of this *in vivo* study demonstrate that peptoid-enhanced lung surfactant replacements exhibit promising bioactivity and can improve physiological and biochemical outcomes using the lung lavage model of ALI. Further investigation is warranted to explore several areas building on the results of this foundational work. Evaluating the bioactivity of different peptoid-based protein mimics (separately and in combination) and lipid carriers, as well as optimization of the surfactant preparation protocol, are important for maximizing the bioactivity of this class of surfactants. It is also important to understand the efficacy of peptoid-enhanced surfactants in alternative animal models including surfactant dysfunction, acid aspiration, endotoxin inhalation, and sepsis-causing cecal ligation and perforation. Lastly, the metabolic fate of peptoids such as these, when delivered to the lung is currently unknown, and it will be important to understand their mechanism of clearance for *in vivo* applications. However, previous studies in which helical, amphipathic peptoids designed to mimic antimicrobial peptides have been used *in vivo* for the treatment of intraperitoneal bacterial infection, have shown them to be well-tolerated and show efficacy in treating infections^62^. A pharmacokinetic study conducted using ^64^Cu-labeled antimicrobial peptoids delivered per oral, intravenously, or intraperitoneally demonstrated that overall the peptoids had higher tissue accumulation, slower elimination, and higher *in vivo* stability compared to peptides^63^.

In conclusion, we have demonstrated for the first time, *in vivo*, that peptoid-enhanced lung surfactant replacements can improve physiological and biochemical outcomes to an extent equivalent to or better than animal-derived surfactant. While all peptoid-enhanced formulations tended to improve outcomes compared to treatment with the lipid carrier alone, **pC** exhibited the best and most sustained *in vivo* response. However, **pB** (in combination with **pC**) will likely still be essential for bioactivity in a successful biomimetic lung surfactant replacement. These promising results demonstrate the potential of peptoid-enhanced surfactants to be functional biomaterials for the treatment of ALI.

## Electronic supplementary material

Supplementary Information
