# Supplementary material for: Effective in vivo treatment of acute lung injury with helical, amphipathic peptoid mimics of pulmonary surfactant proteins
Source: Sci Rep. 2018 May 1;8:6795. doi: 10.1038/s41598-018-25009-3 (PMC5931611; doi:10.1038/s41598-018-25009-3)
Supplement: Supplementary file 1 — Supplementary Information [file 41598_2018_25009_MOESM1_ESM.doc]

**Supplementary Information**

**Effective *in vivo* treatment of acute lung injury with helical, amphipathic peptoid mimics of pulmonary surfactant proteins**

Ann M. Czyzewski, Lynda M. McCaig, Michelle T. Dohm, Lauren A. Broering, Li-Juan Yao, Nathan J. Brown, Maruti K. Didwania, Jennifer S. Lin, Jim F. Lewis, Ruud Veldhuizen, Annelise E. Barron

**METHODS**

**Peptoid Synthesis and Purification – Additional Details**

Peptoids were synthesized using an ABI 433A peptide synthesizer (Foster City, CA) on Rink amide resin (NovaBiochem, San Diego, CA) as described^1^ using peptoid synthesis reagents (Sigma-Aldrich (Milwaukee, WI), solvents (Fisher Scientific (Pittsburgh, PA), and side-chain primary amines benzylamine, octadecylamine, isopropylamine, isobutylamine, S-alpha methylbenzylamine, N-tert-butoxycarbonyl-1,4 diaminobutane, and L-proline. Resin-bound peptoids were cleaved in an trifluoroacetic acid-scavenger mixture and purified by standard reversed-phase HPLC (RP-HPLC) (C4 or C18 column, linear acetonitrile/water gradient). Analytical RP-HPLC purity was > 97%; and molar masses were confirmed using electrospray ionization mass spectrometry (ESI/MS).

**Preparation of Surfactant Mixtures – Additional Details**

Dipalmitoyl phosphatidylcholine (DPPC) and palmitoyoleol phosphatidylglycerol (POPG) (Avanti Polar Lipids, Alabaster, AL), palmitic acid (PA) (Sigma-Aldrich), and solvents (Fisher Scientific) comprised the Tanaka lipid formulation^2^ (22.5 mg Tanaka lipids per animal, enough to treat a 450g rat at 50 mg/kg), which was prepared in a glass vial by combining individual lipid stock solutions to yield a 68:22:9 (w:w:w) mixture of DPPC:POPG:PA in chloroform/methanol (3:1 v:v) solution. Peptoid was added to the lipids from methanol stock solutions at ~ 2 mol% peptoid (~10 wt% relative to total lipid content), and in two-peptoid formulations, 1 mol% per peptoid. Surfactant mixtures were dried under nitrogen, lyophilized, and stored at -20 ^o^C.

**RESULTS**

***Figure S1****.* Vital signs of all animals throughout the timecourse of the experiment. (*A*) Heart rate and (*B*) blood pressure at baseline measurement (BL), after lavage and before exogenous surfactant treatment (Pre-Rx), and at time points throughout the ventilation period. Error bars indicate the standard error of the mean (SEM).

**A**

**B**

**References**

1 Zuckermann, R. N., Kerr, J. M., Kent, S. B. H. & Moos, W. H. Efficient Method for the Preparation of Peptoids [Oligo(N-Substituted Glycines)] by Submonomer Solid-Phase Synthesis. *J Am Chem Soc* **114**, 10646-10647, doi:DOI 10.1021/ja00052a076 (1992).

2 Tanaka, Y. *et al.* Development of synthetic lung surfactants. *J Lipid Res* **27**, 475-485 (1986).
